# Retinal synaptic regeneration *via* microfluidic guiding channels

**DOI:** 10.1038/srep13591

**Published:** 2015-08-28

**Authors:** Ping-Jung Su, Zongbin Liu, Kai Zhang, Xin Han, Yuki Saito, Xiaojun Xia, Kenji Yokoi, Haifa Shen, Lidong Qin

**Affiliations:** 1 Department of Nanomedicine, Houston Methodist Research Institute, Houston, TX 77030, USA; 2 Department of Cell and Developmental Biology, Weill Medical College of Cornell University, New York, NY 10065, USA; 3 Department of Molecular and Cellular Oncology, The University of Texas M. D. Anderson Cancer Center, Houston, TX 77030, USA

## Abstract

*In vitro* culture of dissociated retinal neurons is an important model for investigating retinal synaptic regeneration (RSR) and exploring potentials in artificial retina. Here, retinal precursor cells were cultured in a microfluidic chip with multiple arrays of microchannels in order to reconstruct the retinal neuronal synapse. The cultured retinal cells were physically connected through microchannels. Activation of electric signal transduction by the cells through the microchannels was demonstrated by administration of glycinergic factors. In addition, an image-based analytical method was used to quantify the synaptic connections and to assess the kinetics of synaptic regeneration. The rate of RSR decreased significantly below 100 μM of inhibitor glycine and then approached to a relatively constant level at higher concentrations. Furthermore, RSR was enhanced by chemical stimulation with potassium chloride. Collectively, the microfluidic synaptic regeneration chip provides a novel tool for high-throughput investigation of RSR at the cellular level and may be useful in quality control of retinal precursor cell transplantation.

Retinal degeneration is a leading cause of blindness that, together with glaucoma, retinitis pigmentosa, and age-related macular degeneration, will affect 196 million people worldwide in 2020^1^. The thickness of human retina approximately varies from 100 micron at periphery and 250 micron at optic nerve head and the retina is composed of three layers (from the interior to the exterior surface: the ganglion neurons, bipolar neurons, and photoreceptors). A large number of degenerative retinal diseases are associated with the degeneration of these three layers^2^. To date, there have been few therapeutic options for patients with retinal degeneration. One potential strategy for treatment of this condition is cell transplantation to regenerate retinal tissue. Recent studies have demonstrated the potential of replacing degenerated photoreceptors by injection of either immature rod photoreceptors or engineered stem cells^3^,^4^,^5^. Successful cell therapy based on transplantation of retinal precursors derived from postnatal retinal cells or embryonic stem cells is critically dependent on migration, integration, and maturation of the transplanted cells^6^,^7^. In addition, it has been shown in various mouse eye disease models that the developmental stage of transplanted retinal precursors is important to the success of integration and maturation of donor and host cells^8^,^9^. However, it remains challenge to increase the efficiency of integration and maturation in mouse models as well as in human clinical trials. To date, only a limited number of patients have experienced improved eyesight following transplantation of retinal precursor cells (derived from human embryonic stem cells) into degenerated retinal tissue^5^,^10^. It is still largely unknown how donor and host cells communicate with each other. Therefore, there is a critical need to develop an *in vitro* platform that can mimic retinal function, permit investigation of communication between transplanted and host cells, and facilitate high-throughput screening of retinal regenerative factors at the cellular level to improve the success rate of cell therapy.

Communication between the layered retinal neurons consists of sending and receiving signals through synapses, which are specialized intercellular junctions^11^. *In vitro* reconstruction of synaptic connections is an important approach to investigate retinal neuron communication. Traditionally, the study of retinal intercellular communications has been conducted in petri dishes with a random distribution of cells. However, it is difficult to mimic the layered retinal structure, delineate the topologic effect, control the direction of synapses, and quantify changes in synaptic connectivity. Recently, micro-fabricated channels have been used to guide neuronal growth and generate an organized network of synaptic connections^12^,^13^,^14^, particularly for brain neurons. However, to the best of our knowledge, no study has reported synaptic regeneration of retinal neurons using microfabricated channels with geometric guidance. Therefore, we hypothesize that the designed microchannels might facilitate regeneration of the retinal synaptic connections and reconstruction of the *in vivo* functions.

Here, we present a new method for quantitative analysis of retinal synaptic regeneration (RSR) through image-based counting of connected microchannels in a microfluidic chip (RSR-Chip). In this study, multiple arrays of microchannels with the dimensions 50 or 100 μm long, 3 or 4 μm wide, and 17 μm high were utilized to reconstruct the retinal neuronal synapse and form an organized network using R28 retinal precursor cells. R28 cells are derived from postnatal mouse retina and are a popular model for investigation of retinal cell therapy because they differentiate into photoreceptors, Müller cells, and ganglion cells^3^,^9^,^15^. The length of the microchannels can recapitulate the short-range (50–100 μm) interactions of retinal cells, such as amacrine-bipolar cell interactions^16^. We demonstrate that the synaptic connections of the precursor cells regenerated and formed a network of oriented synapses in the RSR-Chip. The functions of the regenerated synapses were confirmed by immunodetection of phosphorylated extracellular signal-regulated kinase (pERK) of retinal precursors. Furthermore, the effects of inhibitory and excitatory molecules on the dynamics of synaptic regeneration were assessed.

## Results

### Biomimetic retinal synaptic connection

**Figure 1:**
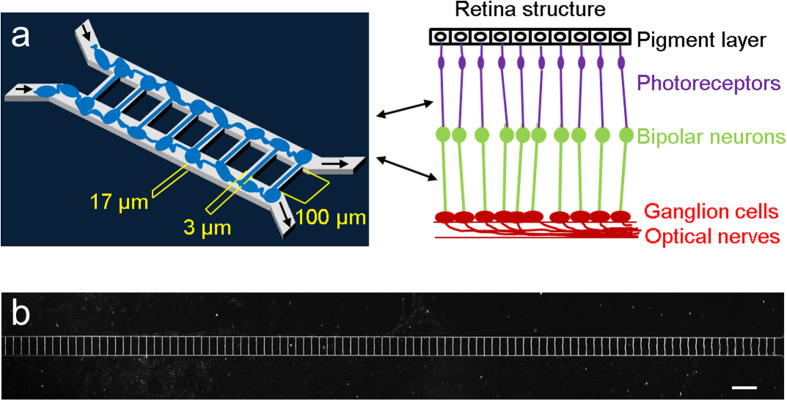
Microfluidic chip design and retinal synapse principle function. (**a**) Design of retinal synapse regeneration (RSR) chip to mimic retinal structure. The RSR-Chip consists of two chambers connected by 100 microchannels. Two populations of retinal cells are seeded in the two chambers, and form synaptic connections in the microchannels. (**b**) Image of microchannels in RSR-Chip. Scale bar, 200 μm.

The retina is a multilayered structure composed of highly specialized and organized neurons. The synaptic connections between two populations of neurons, such as ganglion cells and bipolar neurons, are well aligned, as shown in Fig. 1a. The RSR-Chip was designed to mimic the retinal synaptic connection and features two microchambers connected by multiple arrays of microchannels (Fig. 1b and Supplementary Figure S1). The microchannels are 50 and 100 μm in length, 3 and 4 μm in width and 17 μm in height. We have tried microchannels with different widths and found microchannels with width less than 5 μm can block cell bodies and permit the growth of axon and dendrite. Therefore, we selected channel width 3 μm and 4 μm, and studied whether the two different channel widths show different effect on the reconstruction of synaptic connections. Two populations of retinal cells were seeded separately in the two microchambers (Supplementary Figure S2). Fluid flow was controlled by gravity pressure, and cells of the two populations attached and grew uniformly along two chambers.

**Figure 2:**
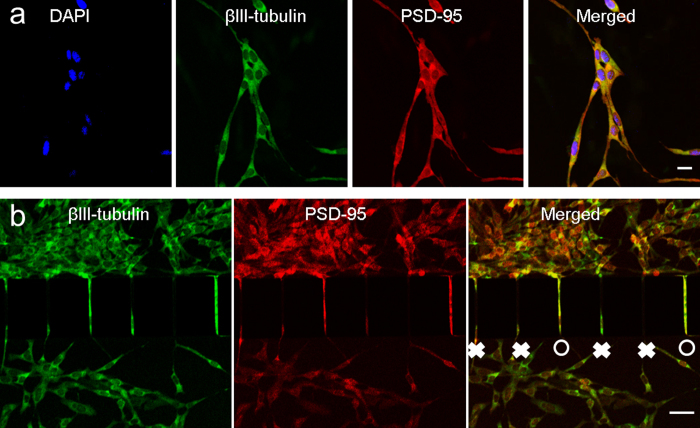
Immunostaining and quantification of retinal synaptic connections. (**a**) Immunostaining of βIII-tubulin and synaptic marker PSD-95 in R28 cells. Scale bar, 50 μm. (**b**) Quantification of synaptic connection by immunostaining of βIII-tubulin and PSD-95. Connections are counted based on the fluorescence microscopic visualization of both βIII-tubulin and PSD-95 in the microchannels. O: synaptic connection. ×: synaptic disconnection. Scale bar, 50 μm.

The R28 retinal precursor cells used in the study are well characterized and differentiate into at least three cell types: retinal ganglion cells, Müller glial cells, and photoreceptors^15^,^17^. Supplementary Figure S3a shows R28 cells were stained with antibody against the ganglion cell biomarker βIII-tubulin. R28 cells also have the expression of Müller glial cells that support neurons and funnel light to the photoreceptors^18^, as shown by immunostaining of vimentin (Supplementary Figure S3b). After 3 days in culture, cells in the two populations extended axons through the microchannels toward the other population in response to secreted cytokine and formed a network of aligned synaptic connections. Synaptic connections were characterized by postsynaptic density protein 95 (PSD-95), which is a synaptic marker protein (Fig. 2a).

### Quantification of retinal synaptic connections

Retinal synaptic regeneration in the RSR-Chip was quantified by counting the precursor cells that are synaptically connected. Connections are counted based not only on occupation of the microchannels by axons but also by fluorescence microscopic visualization of both PSD-95 and βIII-tubulin. Immunostaining of PSD-95 and βIII-tubulin in microchannels (Fig. 2b) clearly reveals synaptic connection (SC) and synaptic disconnection (SD). There are four micro-channels that protrusion of either axon or dendrite protrusions inside the guided micro-channels are observed (labeled as white cross). Furthermore, there are two micro-channels that functional synaptic connections are formed (labeled as white circle) as confirmed by both PSD-95 and βIII-tubulin.

### Dose-dependent induction of functional synaptic communication by glycinergic factors

**Figure 3:**
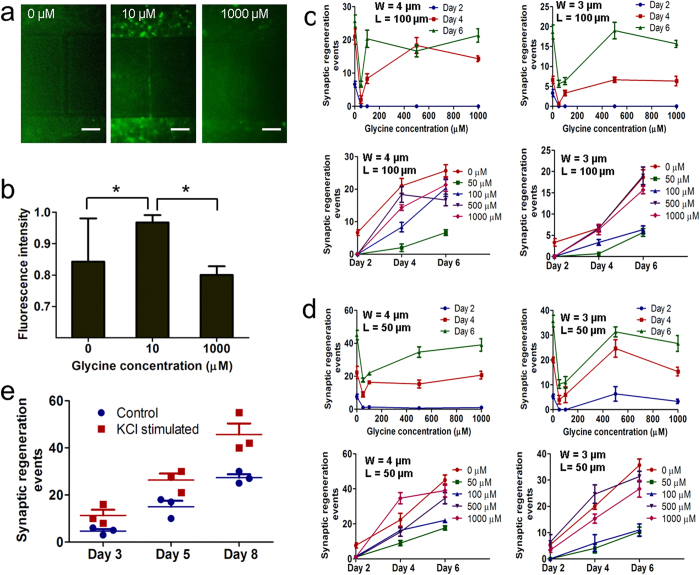
Induction of functional synaptic communication by glycinergic factors and potassium chloride (KCl). (**a**) Immunostaining image of phosphorylated extracellular-related kinase (pERK) in retinal cells treated with glycine at concentrations of 0, 10 and 1000 μM. Scale bar, 50 μm. (**b**) Histogram of fluorescence intensity of pERK and glycine concentrations at 0, 10 and 1000 μM. *p < 0.05. (**c–d**) Effect of microchannel width (4 μm and 3 μm) with channel length of 100 μm (**c**) and 50 μm (**d**) on retinal synaptic regeneration at glycine concentrations of 0, 50, 100, 500 and 1000 μM. W: channel width. L: Channel length. (**e**) Dynamics of chemically induced retina synaptic regeneration via the stimulation of KCl, compared to control sample without KCl treatment on days 3, 5 and 8. The data in b and c represents the mean ± s.e.m. with n = 3.

We next examined the retinal synaptic function of the inhibitory neurotransmitter receptors and determined whether the oriented synaptic network connected through the microchannels was active. Glutamate is a major neurotransmitter transported through the retina via the vertical pathway from photoreceptors to bipolar cells and then to ganglion cells^16^. Electrical stimulation of the glutamatergic retinal signal transduction pathway triggers the phosphorylation cascade that leads to activation of ERK^13^,^19^. R28 cells were treated with glycine, and pathway activity was assessed by fluorescence imaging of pERK. Glycine is one of the major inhibitory neurotransmitters in mammalian retina^20^. R28 cells were treated with 0 μM, 10 μM, and 1000 μM glycine (Fig. 3a). We found that the phosphorylation of ERK was increased by 20% at a low dose of 10 μM compared with the high dose of 1000 μM (Fig. 3b).

We further quantitatively analyzed dose-dependent retinal synaptic regeneration by glycinergic factors through time-lapse counting of synaptic connections. The channel widths of 3 μm and 4 μm and the lengths of 50 μm and 100 μm were specifically designed to accommodate retinal synapse regeneration. Retinal precursor cells were treated with glycine at concentrations of 0, 50, 100, 500, and 1000 μM. The cells were seeded in both chambers at a density of 10^7^ cells/mL and migrated toward the ends of the microchannels. Retinal synaptic connection events in the microchannels at various concentrations of glycine and various microchannel dimensions were imaged and counted on days 2, 4, and 6. First, the effect of channel width (4 μm and 3 μm) with channel length of 100 μm was considered (Fig. 3c). The results show that regeneration events increased within 6 days of culture. Observing the effect of glycine concentration on synaptic regeneration, we found that synaptic regeneration events decreased primarily at glycine concentrations of less than 100 μM. In addition, we considered the effect of varying channel width with the shorter microchannel length of 50 μm on synaptic regeneration (Fig. 3d). We also observed that synaptic regeneration events decreased primarily at lower glycine concentrations of less than 100 μM and approached steady state at higher concentrations. The difference between the effects of microchannel lengths of 50 μm and 100 μm on synaptic regeneration was that the number of regeneration events at high glycine concentration was greater with a length of 50 μm and lower for 100 μm. Overall, it was found that the low concentration of glycine contributed primarily to a decrease in synaptic regeneration, which approached a relatively constant level at high concentration.

### Chemically induced synaptic regeneration

It has been reported that neuronal activity is promoted by chemical stimulation with potassium chloride (KCl)^13^,^14^,^21^. Having modulated the neuronal connections of retinal precursors with inhibitory neurotransmitters, we next investigated whether stimulation with KCl enhanced establishment of retinal synaptic connections. Retinal precursor cells were stimulated with 50 mM KCl for 20 minutes every day. Figure 3e shows the dynamics of chemically-induced synaptic regeneration in the RSR-Chip with microchannel dimensions of 4 μm wide and 100 μm long. KCl stimulation induced an increase in synaptic regeneration events in retinal precursors compared with the control treatment. In particular, KCl-induced regeneration events were increased by approximately 50% compared with control on day 8.

## Discussion

The retinal synaptic connections are regenerated inside the RSR-Chip and form a network of aligned synapses. Based on immunostaining of retinal precursor cells (Fig. 2), the synaptic regeneration events can be easily counted and analyzed in an automated and high-throughput manner. In order to mimic the synaptic integration between two or more populations of retina neurons, such as the interaction of donor retinal precursor cells and host mature retinal cells, the visualization of PSD-95 and βIII-tubulin in retinal precursors in the microchannels was used to quantify synaptic regeneration and serve as an indicator to assess the extent of synaptic integration between two populations.

The electrical communication between retinal precursors through the microchannels was detected by fluorescence imaging of pERK (Fig. 3a). We found that retinal precursors treated with low concentrations of glycine showed stronger pERK signals than those treated with high concentrations of glycine. This observation implies that retinal precursors have more active electrical communication through the microchannels at the lower concentration of 10 μM glycine. It is known that glycine is a major inhibitory neurotransmitter in the retina^20^. A recent study showed that the electric current responses of retinal ganglion cells to glycine at concentrations higher than 100 μM indicated significant desensitization^22^. In our study, retinal precursors also express a biomarker of retinal ganglion cells, as indicated by the staining of βIII-tubulin in Supplementary Figure 3a. Therefore, the glycine receptors of retinal precursors are desensitized. The corresponding level of pERK staining is lower at a higher concentration of 1000 μM glycine, and less electrical signal transduction between precursors through the microchannels is observed. The results of glycine-induced pERK expression in retinal precursors are consistent with the glycine-induced electric current response of retinal ganglion cells^22^. Furthermore, the RSR-Chip demonstrates the capability to recapitulate the electric physiology of the retina via detection of pERK. The RSR-Chip assists the retinal neuronal cells to form not only the structural but also the functional connections by validation of PSD-95 and pERK.

In the experiments, two populations of retinal precursors were loaded into separate chambers connected by microchannels, and the cells formed synaptic connections in response to secreted cytokine. In the shorter 50-μm microchannels, more synaptic regeneration events were observed than in the longer 100-μm microchannels (Fig. 3c–d). It was also found that channel length has a greater impact on regeneration kinetics than channel width. At a constant channel length of either 100 μm (Fig. 3c) or 50 μm (Fig. 3d), similar results were found for channel widths of 4 μm and 3 μm. Our results on the effect of channel dimensions on synaptic connections are consistent with previous reports that elongated microchannel length has significant effect on the kinetics of axonal outgrowth^12^,^13^.

The effect of the inhibitory molecule glycine on synaptic connection was further analyzed. Previous studies indicate that the glycine receptors of retinal ganglion cells approach desensitization at glycine concentrations higher than 100 μM^22^. It was also found that synaptic regeneration events decrease significantly in the concentration range 0–100 μM and approach steady state at higher concentrations (Fig. 3c–d). Therefore, the regenerated retinal synapse behaves in a normal physiological manner, and the RSR-Chip provides an alternative to the traditional approach of single cell electrophysiology to study the effects of inhibitory and excitatory molecules on the dynamics of synaptic regeneration. Further, the neuronal activity of the retinal precursors was chemically stimulated by KCl, and KCl treatment increased synaptic connection events by 50% compared to the control treatment (Fig. 3e). This further demonstrates the biological function of the synapse regenerated inside the RSR-Chip.

The RSR-Chip assists the retinal neuronal cells to form the oriented and functional synaptic connections. Imaging-based analysis demonstrates the quantification of dynamical synaptic regeneration under inhibitor neurotransmitters and induction molecules. In addition to mimicking the retina structure and studying the regeneration of synaptic regeneration, the proposed methodology may also serve an alternative tool to understand the binding dynamics of neuronal receptors. Furthermore, in the field of cell therapy, our method may also serve as quality control of transplant cells and provide the insight into how the transplant cells do integrate and maturate with the host cells and then form the functional synaptic connections.

## Conclusions

In this study, we have developed an RSR-Chip to mimic retinal functions and regenerated retinal synaptic connections in a high-throughput manner. We have shown the regeneration of retinal synaptic connections in the chip and demonstrated that the oriented network of synapses inside the guiding microchannels is physiologically functional. Furthermore, an image-based method was developed to quantify the kinetics of retinal synaptic regeneration in the presence of inhibitory and excitatory molecules. The findings in regard to kinetics of in-chip synaptic regeneration are consistent with the results of conventional electrophysiological analysis at the single-cell level. In addition, we have found that the rate of retinal synaptic regeneration decreases primarily at low concentrations of inhibitory molecules. The integration of a microfluidic chip and a high-throughput image-based method may serve as a valuable tool to investigate the mechanism underlying generation of synaptic connections and screen retinal regenerative factors.

## Methods

### Design and fabrication of microfluidic RSR-Chip

All designs were drawn using AutoCAD and laid out on glass photo-masks. The RSR-Chips were then fabricated using standard photolithography and soft lithography techniques. Briefly, SU-8 3025 photoresist (MicroChem Corp, Newton, MA) was used to fabricate microstructures on silicon wafers 17 μm in thickness. PDMS prepolymer (10A:1B, Sylgard 184 silicone elastomer kit; Dow Corning, Midland, MI) was poured onto the photoresist mold and heated at 80 °C for 45 min. After curing, PDMS was peeled off the photoresist mold and holes were punched for the inlets and outlets. The PDMS layer (0.4 mm in thickness) was laid on the cell culture dish (Corning) and exposed to vacuum for 15 min before the experiment. Because PDMS is hydrophobic, the media can fulfill the microchannel without leakage between PDMS and dish. We have tested the chip with red dye solution for two weeks and found the solution was stable, which demonstrates the bond between PDMS and dish is strong enough to achieve fluidic isolation.

### Retinal cell culture

The R28 immortalized retinal precursor cell line (Kerafast, Boston, MA) were derived from postnatal day 6 Sprague-Dawley rat retina, immortalized by expression of the adenovirus 12S E1A gene, and expressed retinal ganglion cell, Müller glial cell, and photoreceptors^15^. R28 cells were cultured in Dulbecco’s modified Eagle medium supplemented with 10% (v/v) fetal bovine serum (FBS) and 1% penicillin-streptomycin. All cells were cultured at 70% humidity with 5% CO_2_ at 37 °C.

### On-chip loading and culture of retinal precursor cells

R28 cells were dissociated and used at a density of 10^7^ cells/mL for all experiments in the study. Two populations of precursor cells, each in a volume of 80 μL, were loaded at the two inlets and separated in two independent microfluidic chambers without mixing. Fluid perfusion was controlled by gravity pressure. After one day of culture, the cells in the two populations spread uniformly and attached along the two individual chambers. Culture medium was replaced daily. After three days in culture, cells in each population extended axons through the microchannels toward the other population in response to diffusion of secreted cytokines and formed a network of aligned synaptic connections. To study the effect of glycinergic factors on retinal synaptic regeneration, cells were cultured in medium containing 10 μM, 50 μM, 100 μM, 500 μM, or 1000 μM glycine. To study the effect of stimulation with potassium chloride (KCl) on retinal synaptic connections, cells were stimulated with 50 mM KCl for 20 minutes every day. Control experiment was done without KCl stimulation.

### Immunocytochemistry

Cells were fixed with 4% paraformaldehyde at room temperature for 20 min and stained for the presence of vimentin , βIII-tubulin and PSD-95. Vimentin is a biomarker for retinal glia, Müller glia, and astrocytes^23^. βIII-tubulin is a phenotypic biomarker for somata of retina ganglion cells^24^. PSD-95 is a postsynaptic marker. After fixation, cells were incubated in phosphate-buffered saline (PBS) containing 0.1% TritonX-100 for 45 min and then blocked in 3% bovine serum albumin for 30 min at room temperature. Cells were washed three times with PBS for 30 min, incubated with anti-βIII-tubulin (1:200, Abcam, Cambridge, UK) and anti-PSD-95 (1:200, Abcam) overnight at 4 °C. Cells were washed twice with PBS for 10 min and incubated with Alexa Fluor 488 and Alexa Fluor 594-conjugated secondary antibodies for 1 h at room temperature. Phosphorylated ERK of the electric signal transduction pathway was stained using antibody *p*-ERK-AF488 (SC-7383; Santa Cruz Biotechnology, Santa Cruz, CA) at a dilution of 1:100.

### Image acquisition

Retinal precursor cells were imaged using a FluoView™ 1000 inverted confocal microscope (Olympus). For counting of synaptic regeneration events and time-lapse phase contrast and fluorescence imaging, an EVOS microscope (Life Technologies, Grand Island, NY) was used with environmental controls set at 5% CO_2_, 37 °C, and 70% humidity.

### Statistical analysis

For the analysis of synaptic connections and phosphorylated ERK, data from three independent experiments were assessed using an unpaired Student’s *t*-test. For all analyses, *p *< 0.05 was considered statistically significant.

## Additional Information

**How to cite this article**: Su, P.-J. *et al.* Retinal synaptic regeneration *via* microfluidic guiding channels. *Sci. Rep.* **5**, 13591; doi: 10.1038/srep13591 (2015).

## Supplementary Material

Supplementary Information
