# Supplementary material for: Retinal synaptic regeneration via microfluidic guiding channels
Source: Sci Rep. 2015 Aug 28;5:13591. doi: 10.1038/srep13591 (PMC4551994; doi:10.1038/srep13591)
Supplement: Supplementary Information [file srep13591-s1.pdf]

Supplementary materials

Retinal synaptic regeneration *via* microfluidic guiding channels

Ping-Jung Su[1,2*], Zongbin Liu[1,2*], Kai Zhang[1,2], Xin Han[1,2], Yuki Saito[1], Xiaochun Xia[1], Kenji Yokoi[1], Haifa Shen[1], Lidong Qin[1,2,3]

[1]Department of Nanomedicine, Houston Methodist Research Institute, Houston, TX 77030, USA
[2]Department of Cell and Developmental Biology, Weill Medical College of Cornell University, New York, NY 10065, USA
[3]Department of Molecular and Cellular Oncology, The University of Texas M. D. Anderson Cancer Center, Houston, TX 77030, USA

Correspondence and requests for materials should be addressed to L.Q. (LQin@houstonmethodist.org).
[*] These authors contributed equally to this work.

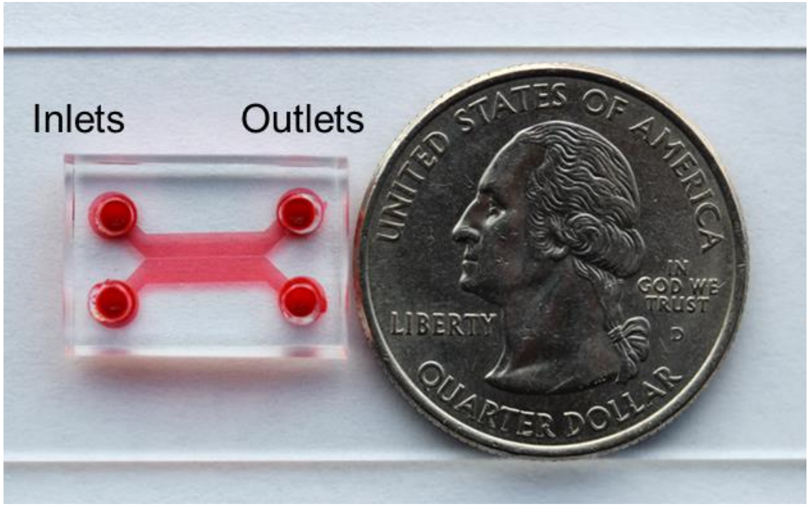

Figure S1 | Photo of retinal synaptic regeneration chip.

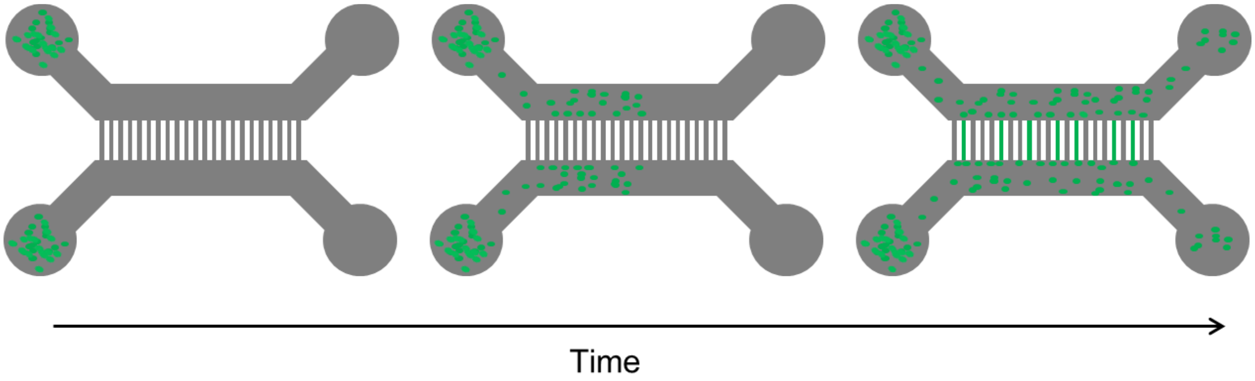

Figure S2 | Procedure of loading retina precursor cells. Loading cells through the chamber is controlled by gravitation pressure.

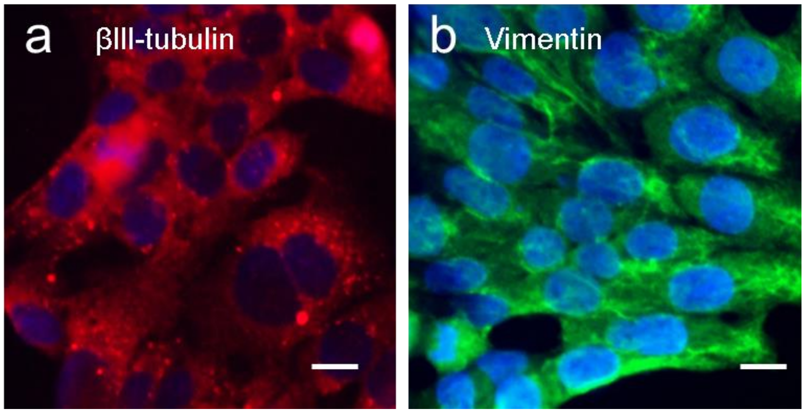

Figure S3 | Immunostaining of βIII-tubulin as the biomarker of retinal ganglion cells and vimentin as the biomarker of Müller cells. Scale bar, 20 μm.
